# Influence of a New “Call-Out Algorithm” for Management of Postoperative Pain and Its Side Effects on Length of Stay in Hospital: A Two-Centre Prospective Randomized Trial

**DOI:** 10.1155/2017/9431984

**Published:** 2017-08-10

**Authors:** Lisa Dybvik, Erlend Skraastad, Aigerim Yeltayeva, Aidos Konkayev, Tatiana Musaeva, Igor Zabolotskikh, Lars Bjertnaes, Vegard Dahl, Johan Raeder, Vladimir Kuklin

**Affiliations:** ^1^Department of Anaesthesia and Intensive Care, Akershus University Hospital, 1478 Lørenskog, Norway; ^2^Department of Anaesthesia and Intensive Care Medicine, Kongsberg Hospital, 3612 Kongsberg, Norway; ^3^Institute of Clinical Medicine, University of Oslo, 0316 Oslo, Norway; ^4^Department of Anaesthesia and Intensive Care, Kazakh State Medical University, Astana, Kazakhstan; ^5^Department of Anaesthesia and Intensive Care, Scientific Research Institute of Traumatology and Orthopaedics, Astana 01005, Kazakhstan; ^6^Department of Anaesthesia and Intensive Care, Kuban State Medical University, Krasnodar 350063, Russia; ^7^Anaesthesia and Critical Care Research Group, Department of Clinical Medicine, Faculty of Health Sciences, The Arctic University of Norway (UiT), 9037 Tromsø, Norway; ^8^Department of Anaesthesia and Intensive Care Medicine, Oslo University Hospital and Institute of Clinical Medicine, Medical Faculty, University of Oslo, 0316 Oslo, Norway

## Abstract

**Background:**

We recently introduced the efficacy safety score (ESS)* as* a new “call-out algorithm” for management of postoperative pain and side effects. In this study, we report the influence of ESS recorded hourly during the first 8 hours after surgery on the mobility degree, postoperative nonsurgical complications, and length of hospital stay (LOS).

**Methods:**

We randomized 1152 surgical patients into three groups for postoperative observation: (1) ESS group (*n* = 409), (2) Verbal Numeric Rate Scale (VNRS) for pain group (*n* = 417), and (3) an ordinary qualitative observation (Control) group (*n* = 326). An ESS > 10 or VNRS > 4 at rest or a nurse's observation of pain or adverse reaction to analgesic treatment in the Control group served as a “call-out alarm” for an anaesthesiologist.

**Results:**

We found no significant differences in the mobility degree and number of postoperative nonsurgical complications between the groups. LOS was significantly shorter with 12.7 ± 6.3 days (mean ± SD) in the ESS group versus 14.2 ± 6.2 days in the Control group (*P* < 0.001).

**Conclusion:**

Postoperative ESS recording in combination with the possibility to call upon an anaesthesiologist when exceeding the threshold score might have contributed to the reductions of LOS in this two-centre study. This trial is registered with NCT02143128.

## 1. Introduction

The aim of modern management of postoperative pain is to enable functioning while relieving suffering; it is not enough to minimize side effects. Still, between 20% and 40% of surgical patients report high levels of postoperative pain, and almost 25% have experienced adverse effects of opioid analgesics [1]. Unsatisfying methods for evaluation of efficacy and side effects of analgesics, irregular recording of clinical information, and absence of a clearly defined “call-out algorithm” for nurses might contribute to a postoperative pain treatment suffering from both side effects and fatalities [1–5]. A large study performed in four New York hospitals revealed that patients with higher pain scores at rest had significantly longer length of hospital stay (LOS) [6]. According to the latter investigators, moderate-to-severe pain at rest and reduced mobility after surgery were associated with increase neither in complications nor in morbidity and mortality postoperatively. The authors suggest that improved pain control might reduce LOS [6].

Other investigators recently reported that side effects of drugs had more than doubled in the hospitals that had introduced pain management guided by the patients' own numerical scale scores [7]. These authors suggested that more than just a one-dimensional numeric assessment of pain should be surveyed to make postoperative treatment safe and effective [7]. We developed the efficacy safety score (ESS), a new “call-out algorithm” for nurses in surgical departments, and implemented it in clinical practice at Kongsberg Community Hospital in Norway [8] after reports of fatalities due to postoperative overdoses of opioids in Norwegian hospitals [4, 5]. We established ESS after obtaining consensus in a DELPHI process between 10 international experts [9] on which parameters should be included in the score. The final version of ESS consists of the sum of two subjective parameters (Verbal Numeric Rating Scale at rest and during mobilization) and four vital parameters (consciousness levels, postoperative nausea/vomiting, circulation, and respiration status), as depicted in Table 1 [8]. The mathematical sum of ESS ≥ 10 is agreed upon by the experts as “the call-out alarm level” for informing the anaesthetist on duty, while any single score of 15 (on either consciousness, circulation, or respiration) is proposed as an alarm limit for immediate support of the patient [8]. Subsequently, we validated ESS for score criteria quality [10] and sensitivity for reflections of the patient's postoperative status [8]. Many factors like type of surgery, postoperative pain monitoring, and treatment can influence LOS [6], but, thus far, the topic has been undercommunicated. We hypothesized that better control of postoperative pain treatment and its side effects by monitoring ESS might influence the degree of mobility and morbidity in surgical patients and consequently reduce LOS. Thus, our aim was to validate the influence of recording ESS and the application of a “call-out algorithm” on LOS in two university hospitals in which the routine policy of registration of pain as “the fifth vital sign” had not been adopted yet. The primary endpoint of the study was to assess LOS in groups of patients with different types of clinical data records and “call-out algorithms,” while secondary endpoints were to compare the degree of mobilization, number of postoperative nonsurgical complications, and 28-day survival between the groups.

## 2. Methods

### 2.1. Ethics

Ethical approval of this clinical trial was provided by the Ethical Committee of Scientific Research Institute of Traumatology and Orthopaedics, Astana, Kazakhstan (ref. 2014-002, Chairperson: Professor T. Anashev), on 28 February 2014 and the Ethical Committee of Kuban State Medical University, Krasnodar, Russia (ref. 2014-027, Chairperson: Professor E. Bolotova) on 20 March 2014. In both countries, the study was considered as a quality assessment of efficacy and safety of pain treatment without any intervention apart from enforced surveillance. Thus, the project was approved with no need for informed consent of the patients.

### 2.2. Settings

The study was performed in the departments of abdominal surgery, orthopaedics, gynaecology, urology, and vascular surgery and high dependency units (HDU) at Astana University Hospital, Astana, Kazakhstan, and Krasnodar University Hospital, Krasnodar, Russia.

### 2.3. Inclusion Criteria

During the period from 1 March 2014 to 31 May 2015, all surgical patients whom we expected to need observation in hospital for more than 8 hours postoperatively and were able to communicate adequately with the nursing staff immediately after surgery were considered for inclusion.

### 2.4. Exclusion Criteria

We excluded patients below 18 years of age, patients with poor communication capabilities due to psychiatric diseases, dotage, and language problems, and patients who refused to communicate.

### 2.5. Procedures

**Figure 1:**
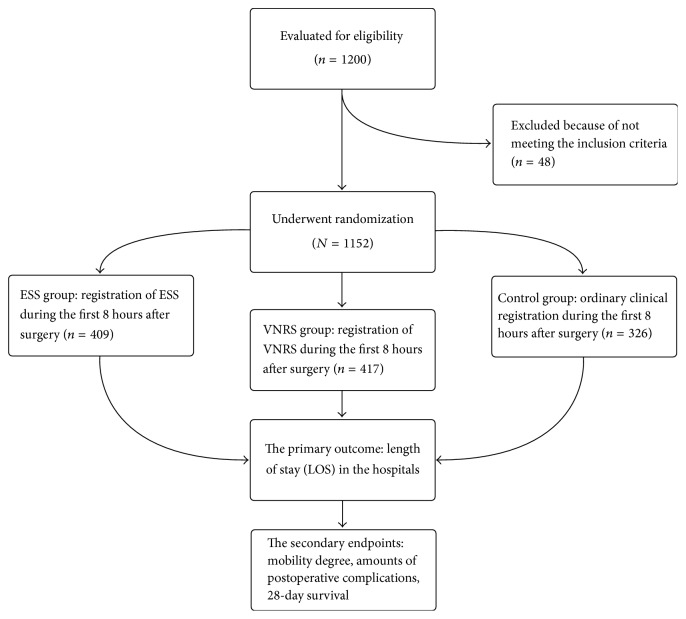
Flow chart of the study.

Figure 1 depicts a detailed plan of the study. After inclusion, we randomized patients (by means of sealed envelopes) into one of three groups: (1) a record of ESS group (ESS group, Table 1), (2) a record of pain with Verbal Numeric Rate Scale group (VNRS group), in which 0 indicates no pain and 10 indicates “worst imaginable pain,” and (3) a group in which ordinary clinical documentation was performed during the first 8 hours after surgery (Control group). In all groups, we recorded the mobility degree hourly during the first 8 hours postoperatively and noticed the degree of mobility from 0 to 3, where 0 indicates lack of mobility, 1 indicates mobilization in bed, 2 indicates mobilization to a chair (bedside), and 3 indicates mobilization to standing. Table 1 presents detailed information about ESS with weighted scores. Based on the results of our study conducted at Kongsberg Community Hospital, Kongsberg, Norway, “call-out” alarm for ESS ≥ 10 was established for consultation by telephone or visit by the responsible anaesthesiologist or acute pain team on duty [8]. In the VNRS group, we based “call-out” decision on VNRS > 4 at rest, while in the Control group “call-out” decision was based on judgement of the patient's clinical condition by a nurse. We defined the ordinary evaluation by nurses in the Control group as the traditional routine clinical observation and care that were usually applied in these hospitals. Nurses in surgical departments and high dependency units recorded all clinical variables and mobility degree, while research fellows collected all demographic variables. The latter also registered all postoperative nonsurgical complications, such as cardiovascular (arrhythmias, ischaemic heart attacks, cardiac failure, low arterial blood pressure, and deep vein thrombosis) and pulmonary symptoms (atelectasis, pleural effusion, pneumonia, and pulmonary embolism) during the first 8 hours after surgery and contacted all patients or their relatives by mobile telephone for verification of 28-day survival. For evaluation of the physiological status of the patients, we used the American Society of Anaesthesiologists (ASA) Classification System. In that system, ASA I depicts a normal healthy patient, ASA II depicts a patient with mild systemic disease without substantial functional limitations, ASA III depicts a patient with one or more moderate-to-severe diseases and substantive functional limitations, ASA IV depicts a patient with severe systemic disease that is a constant threat to life, ASA V depicts a moribund patient who is not expected to survive without the operation, and ASA VI depicts a declared brain-dead patient whose organs are being removed for donor purposes.

### 2.6. Sample Size Calculation

We planned to recruit 180–200 patients into each group during a period of 12 months in each of the participating hospitals (total number: 1080–1200 patients) assuming an 18–20% difference in LOS between the groups for testing of sample size with 80% power and a two-sided significance level of 5%.

All subjective and objective clinical data were recorded in an especially designed program for mini iPad. Five mini iPads in each hospital were used for sampling and registration of clinical data that were subsequently transferred to the Structured Query Language (SQL) database using Clouds technology. Information about the record program with detailed video instructions is available on the following web site: http://essdb.no/index.php/en/application-en.

### 2.7. Statistical Analysis

Statistical data analyses were performed with cluster analyses of intracluster correlation coefficient, one-way ANOVA, and Chi square analyses using IBM® SPSS® Statistics 21.0. Data distribution was assessed using Shapiro-Wilk test. We used Kruskal-Wallis One-Way Analysis of Variance on Ranks to compare the difference between groups. If *F* value was greater than the critical value, ANOVA was followed by Dunn's method for pairwise multiple comparisons to obtain *P* values between groups. The data are presented as means ± standard deviations (SD) for age, Body Mass Index (BMI) as numbers and percentages (*n*, %) for the ESS values, gender, American Society of Anaesthesiologists (ASA) physical status classification, and type of surgery and anaesthesia. The results of hospital length of stay (LOS) in days are presented as median (solid line), mean (dashed line), and 10th, 25th, 75th, and 90th percentiles as vertical boxes with error bars; outliers are presented as open circles.

Additionally, we retested the “null hypothesis” by removing patients with extreme values of LOS from the data analysis. In this analysis, we defined patients with LOS below the 5th percentile and above the 95th percentile of the median as outliers and removed them from the LOS data of each hospital, the clustered LOS data of both hospitals, and the LOS data of all patients after laparoscopic cholecystectomy. Subsequently, we used the Kruskal-Wallis One-Way Analysis of Variance on Ranks to compare the difference between the groups. If *F* value was greater than the critical value, ANOVA was followed by Dunn's method for pairwise multiple comparisons to obtain *P* values between the groups. *P* < 0.05 was regarded as statistically significant.

## 3. Results

Totally, 1152 patients, 679 from the University Hospital of Astana and 473 from the University Hospital of Krasnodar, were included in the study during the period from 3 March 2014 to 26 May 2015. Tables 2 and 3 display basic demographic, anthropometric, and clinical characteristics of the three groups of patients studied in each of the hospitals. As depicted in Table 2, there were no statistically significant differences between the groups in demographic and clinical variables, such as age, BMI, gender, ASA classification, and type of anaesthesia and surgery in patients included in the study at the University Hospital of Astana. In contrast, we found significant differences between the groups in such clinical variables as ASA classification (*P* < 0.0001), type of surgery (*P* = 0.0008), and anaesthesia (*P* = 0.0034) at the University Hospital of Krasnodar. As shown in Table 3, 25.5% of the patients in the Control group were classified as ASA I versus 4.4% in the ESS group. Moreover, we listed 21.6% of the patients of the Control group as ASA III versus 39.7% in the ESS group. Concerning type of surgery, there were differences between the Control and the ESS groups in endocrine surgery (4.9% versus 16.0%) as well as in urological (15.6% versus 7.1%) and vascular (7.8% versus 2.7%) surgery, respectively (Table 3). In the Control group, more patients received spinal anaesthesia as compared with the ESS group (13.7% versus 3.8%). In the former group, more patients also were given total intravenous anaesthesia as compared to the ESS group (4.9% versus 1.6%). Finally, Table 3 also shows that general anaesthesia with sevoflurane and fentanyl was applied more often in the ESS group as compared with the Control group (55.2% versus 37.2%).

**Figure 2:**
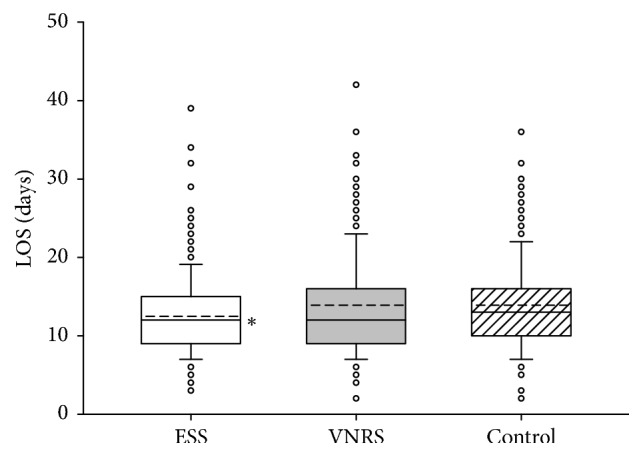
Length of hospital stay (LOS) of patients included in the study in University Hospital of Astana, Astana, Kazakhstan (*n* = 679). Data are presented as vertical boxes with median (solid line), mean (dashed line), and interquartile range with 10th percentile and 90th percentile error bars. Outliers are presented as open circles. ^*∗*^*P* = 0.011 comparing ESS group versus Control group.

**Figure 3:**
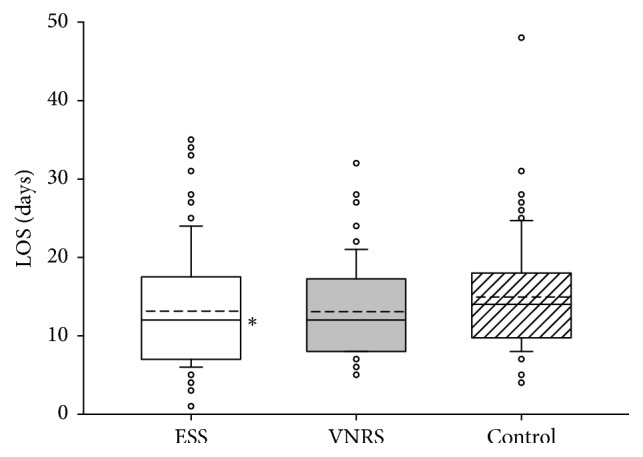
Length of hospital stay (LOS) of patients included in the study in University Hospital of Krasnodar, Krasnodar, Russia (*n* = 473). Data are presented as vertical boxes with median (solid line), mean (dashed line), and interquartile range with 10th percentile and 90th percentile error bars. In the VNRS group, the 10th percentile error bar is matching with the lower line of the box. Outliers are presented as open circles. ^*∗*^*P* = 0.022 comparing ESS group versus Control group.

**Figure 4:**
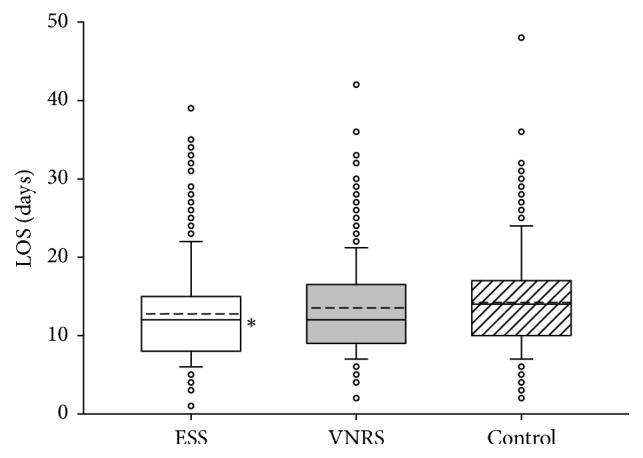
Length of hospital stay (LOS) of patients included in the study from both hospitals (*n* = 1152). Data are presented as vertical boxes with median (solid line), mean (dashed line), and interquartile range with 10th percentile and 90th percentile error bars. Outliers are presented as open circles. ^*∗*^*P* < 0.001 comparing ESS group versus Control group.

We observed no significant differences between the groups and hospitals concerning the degree of mobilization, the number of postoperative nonsurgical complications, or mortality during the 28 days of observation time (data not presented). As depicted in Figures 2 and 3, in both hospitals, patients in the ESS group had significantly shorter LOS as compared to the Control group. Calculation of intracluster correlation coefficients revealed no significant differences in clustered data. Therefore, we pooled the results from both hospitals for further analyses of LOS which demonstrated a significant intergroup difference in LOS between 12.7 ± 6.3 days in the ESS group and 14.2 ± 6.2 days in the Control group (*P* < 0.001) but not between the ESS and the VNRS group: 13.5 ± 6.2 days (Figure 4).

In the ESS group, we found that 120 out of 409 patients (approximately 29%) were registered with ESS of more than 10 after the first postoperative hour, and therefore a telephone consultation or visit by the anaesthesiologist on duty was required according to the “call-out alarm” routine (ESS ≥ 10). However, the number of patients with ESS above 10 decreased gradually during the entire postoperative period, and, at 8 hours postoperatively, only 3.6% (*n* = 15) of the patients had ESS ≥ 10. In total, 517 visits were registered during the first 8 hours of observation in patients with ESS ≥ 10, whereas 4.4% (*n* = 23) were caused by “false alarms,” according to the journal notes of the anaesthesiologists on duty. Correspondingly, in the VNRS and the Control groups, anaesthesiologist made 678 and 296 visits, respectively, whereas 4.7% (*n* = 32) and 2.3% (*n* = 7), respectively, were “false” according to the visiting anaesthesiologists.

**Figure 5:**
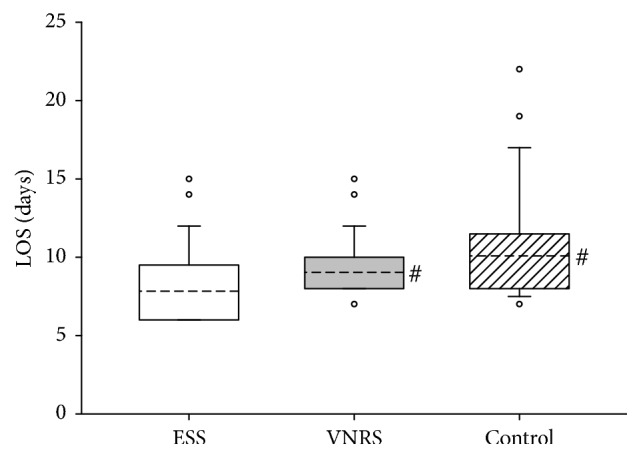
Length of hospital stay (LOS) of patients included in the study after laparoscopic cholecystectomy from both hospitals (*n* = 114). Data are presented as vertical boxes with mean (dashed line) and interquartile range with 10th percentile and 90th percentile error bars. In the ESS and VNRS groups, the 10th percentile error bar is matching with the lower lines of the boxes. Outliers are presented as open circles. ^#^ESS group versus Control group, *P* = 0.003; ^#^ESS group versus VNRS group, *P* < 0.001.

In order to exclude the influence of different types of surgery, we carried out an analysis of a subgroup of 114 patients who underwent laparoscopic cholecystectomy.  Table 4 displays the demographic and clinical characteristics of the patients. We did not find any significant differences between the three groups in such demographic or clinical characteristics as age (*P* = 0.15), gender (*P* = 0.61), or ASA classification (*P* = 0.39) (Table 4). Further, there were no differences between the groups in degree of mobility and number of postoperative nonsurgical complications (data not shown). However, as far as LOS after laparoscopic cholecystectomy is concerned (Figure 5), we observed significantly lower LOS in the ESS group versus the Control group (*P* = 0.003) and in the ESS group versus the VNRS group (*P* < 0.001).

Record of ESS during the first 8 hours after laparoscopic cholecystectomy demonstrated that almost 30% (*n* = 11) of the patients (*n* = 36) had an ESS ≥ 10 at 1st postoperative hour, and according to the “call-out algorithm” they either had a telephone consultation or were seen by the anaesthesiologists on duty. At the end of ESS registration 8 hrs postoperatively, only two patients had an ESS ≥ 10.

In the additional analysis of data from the University Hospital of Astana, we found totally 54 patients with LOS values below and above the 5% and 95% range of the median, respectively. These data were removed from their respective groups. Thus, we removed 19 patients from the ESS group, 15 patients from the VNRS group, and 20 patients from the Control group. Actually, the difference in LOS between the ESS and Control groups remained significant (*P* = 0.003 versus *P* = 0.011). Correspondingly, in LOS data collected at the University Hospital of Krasnodar, we omitted 10, 6, and 7 patients, respectively, from the ESS, VNRS, and Control groups. Intergroup comparison of LOS revealed that differences between the ESS and the Control groups remained significant (*P* = 0.002 versus *P* = 0.022) after omitting the patients with extreme values of LOS. After reanalysing the data, we also found a significantly lowered LOS in the VNRS group in comparison with the Control group (*P* = 0.012).

In the clustered data from both hospitals, we omitted 27, 36, and 28 patients, respectively, from the ESS, VNRS, and Control groups. The significant difference between the ESS and the Control groups (*P* < 0.001) was confirmed, and, additionally, we found a significant difference between the ESS and VNRS groups (*P* = 0.011). Finally, we also confirmed the significant difference between the ESS and the Control groups (*P* = 0.003) after omitting the patients with extreme values of LOS after laparoscopic cholecystectomy.

## 4. Discussion

The main finding of the present two-centre trial was that the length of stay in hospital was significantly lower in the ESS group as compared with the Control group, while we noticed no differences between the VNRS group and the Control group. Correspondingly, in both hospitals, subgroup of patients who underwent laparoscopic cholecystectomy had significantly shorter LOS in the ESS group as compared with the VNRS and the Control groups. Additional statistical analysis revealed that the differences between the ESS and the Control groups, separately in each hospital, and in the clustered data from both hospitals remained significant after omitting the patients with extreme values of LOS. The reanalysis of data also confirmed the significant difference between the ESS and the Control groups in patients after laparoscopic cholecystectomy.

A policy for routine medical record of pain, as “the fifth vital sign,” has not yet been adopted by the University Hospital of Astana in Kazakhstan and University Hospital of Krasnodar in Russia. So far, the latter institutions make no routine use of any postoperative pain or quality assessment score, like the Numeric Rate Scale (NRS) or the Modified Early Warning Score (MEWS), which have different objectives [11]. Consequently, there was no ethical problem for the medical staff of these institutions associated with the inclusion of patients into the Control group of the present study. Most advantageously, the trial was uninfluenced by any other pain score or “call-out algorithm” that could warn against emerging deterioration of patients' wellbeing or general condition. In order to improve the quality of the recorded data, we developed a special program for iPad with alarms that both reminded the nursing staff on the time of data acquisition and alerted the anaesthesiologists on duty in situations with ESS ≥ 10 or VNRS > 4 at rest. In order to avoid any bias, nurses not involved in the study collected the data in this trial. Actually, due to the simplicity in use and the popularity among the nursing staff, the administration of Kongsberg Hospital approved ESS as a method for assessment of efficacy and safety of pain treatment. Detailed information about ESS and the special program for registration on iPad is available on http://esscore.org/ and http://essdb.no/. We primarily tested ESS in a study that included 207 postoperative patients and validated the score against quality criteria proposed for measurement properties of health status questionnaires [10]. Retrospectively, we realize that the latter validation had several biases. Unfortunately, almost 97% of the patients received total intravenous and/or spinal/epidural/regional anaesthesia, and only 3% were anaesthetised with inhalational anaesthetics [8]. In contrast, in Krasnodar and Astana, most of the patients included were anaesthetised with sevoflurane and fentanyl. However, in spite of differences in type of anaesthesia and surgery, we found ESS ≥ 10 at 1st hour postoperatively in 29.3% of the patients. This was nearly the same percentage (29%), at the same time point, as in patients included in the validation study conducted at Kongsberg Hospital. This consistency is in agreement with a previously published clinical study [2] demonstrating that approximately 30% of all surgical patients suffered severe postoperative pain.

On average, pooled data from the two hospitals showed that LOS varied between 12 and 14 days in both hospitals. This is consistent with previously reported health data in Organisation for Economic Co-operation and Development (OECD) [12] demonstrating that the average LOS in Russia is approximately 13.6 days. However, according to European statistics published on the Internet [13], the average length of hospital stay for inpatients ranged from 5.5 days in Bulgaria to 9.6 days in Croatia, with Finland topping the list with an average LOS of 10.6 days. Today, LOS is often used as an indicator of hospital efficiency [13]. Nevertheless, too short average of LOS might cause negative effects on health outcomes [14]. A retrospective study representing three hospitals in Japan and two in the USA demonstrated that median LOS in hip fracture patients was 34 days in Japan and 5 days in the USA [14]. Meanwhile, survival rate at follow-up, six months after surgery, was 89.5% in Japan and 77.2% in USA. Moreover, a Cox regression analysis revealed that every 10-day increase in LOS after surgery was associated with a 26% reduction in the risk of mortality (hazard ratio = 0.744, *P* = 0.014) after adjusting for LOS before surgery, patients' basic characteristics, number of complications, and country. Based on these findings, the authors concluded that shorter LOS after surgery did not necessarily predict better survival rate [14]. The recently published EuroHOPE study that included 59 605 hip fracture patients across seven European countries demonstrated that Hungary had the lowest LOS (12.7 days) and the highest one-year mortality (mean: 39.7%), whereas Italy had the highest mean LOS (23.3 days) and the lowest one-year mortality rate (mean: 19.1%) [15]. Thereto, a cohort study from Sweden, which included 116 111 patients with hip fractures, reported that shorter LOS was associated with increased risk of death 30 days after discharge from hospital but only among patients with LOS 10 days or less [16]. In contrast to the Swedish findings, a cohort study from the USA, which included a total of 188 938 hip fracture patients and a LOS of 11–14 days, was associated with 32% increased odds of death 30 days after discharge, as compared with a LOS of 1 to 5 days (odds ratio: 1.32) [17]. Large differences in the perioperative and postoperative care of hip fracture patients between Japan, Europe, and USA might give the opposite results [17]. Therefore, caution should be exercised when comparing results of this kind of studies between countries with dissimilar health care systems [17].

Our study has several limitations. We found no differences between the three groups in degree of mobility, number of postoperative nonsurgical complications, and 28-day survival. Actually, these findings were not surprising as modern anaesthesia [18] and postoperative analgesia techniques [19] principally demonstrate low incidences of postoperative complications and, consequently, low postoperative morbidity and mortality. However, we were not able to confirm the hypotheses that better monitoring of postoperative pain treatment and its side effects by assessment of ESS could have positive influence on the degree of mobility and, consequently, on the morbidity in surgical patients. Thus, the mechanisms causing the reduction of LOS in the ESS groups remain unknown. Another limitation of our study is that we did not record the total time and dosage of anaesthesia during surgery, and it is unclear whether they were comparable across all participants. We noticed no significant differences between the groups with regard to demographic variables as age, BMI, gender, ASA classification, and type of anaesthesia in the hospital of Astana. In contrast, we found significant differences between the groups regarding ASA classification, type of surgery, and anaesthesia in the University Hospital of Krasnodar. Indeed, these differences might have influence on the length of stay in University Hospital of Krasnodar and, consequently, be considered as a limitation of the study. In order to avoid the effect of differences in ASA classification, type of surgery, and anaesthesia on LOS, we selected and analysed additional data from all patients operated with laparoscopic cholecystectomy in the two hospitals. We found that LOS after laparoscopic cholecystectomy was significantly shorter in the ESS group as compared with the Control group. As mobility degree and morbidity displayed no significant intergroup differences, we could not identify the precise mechanism that contributed to the reduction of LOS in the ESS group. The latter, together with a lack of blinding procedures, also can be considered as limitations of the study. However, it is important to stress that surgeons responsible for the discharge of patients were neither involved in the study nor informed about the primary endpoint of the clinical trial. Thus, we do believe that the medical staffs were sufficiently blinded to exclude any personal influence on the results of the study. In turn, the long average LOS in these hospitals can be partly explained by the fact that, in ordinary clinical practice in Kazakhstan and Russia, patients usually are admitted to hospital 1–4 days prior to surgery for different types of routine investigations, such as blood analyses and preoperative examination by the anaesthesiologist. Taking this into account, “real” LOS in Astana and Krasnodar hospitals might be close to that in Finland with an average of 10.6 days [13].

Finally, we believe that the university hospitals in Krasnodar and Astana have a great potential for reduction of LOS by introduction of such measures as multimodal fast-track programs for surgery [20], day case surgery for laparoscopic cholecystectomy [21], and home health care and institutional long-term care for patients who require additional services [22]. We also hope that the results of our study will inspire the administrators of the hospitals to introduce postoperative quality assessment scores like VNRS, MEWS, or ESS in routine clinical practice.

## 5. Conclusions

Registration of ESS hourly during the first 8 hrs after surgery and the extra attention of the anaesthesiologist on duty might have contributed to the significant reduction of LOS in both hospitals in this two-centre study. Since mobility degree and morbidity were not different between the groups, we could not identify the exact mechanisms behind the reduction of LOS in the ESS group. Consequently, elucidation of the impact of ESS on the length of stay in hospital after various types of surgery will need further randomized controlled trials.

## Figures and Tables

**Table 1:** Description of efficacy safety score (ESS).

	Score
*Mental status*	
Awake and alert patient	0
Awake patient but influenced by drugs; difficulties in communication	5
Acutely confused, upset/uneasy, hallucinated, or euphoric patient	10
Unresponsive patient	15^**∗**^

*Postoperative nausea and vomiting (PONV) status*	
No postoperative nausea and vomiting	0
Postoperative nausea only	5
Postoperative nausea and vomiting/retching	10

*Pain status at rest*	
No postoperative pain	0
Low-intensity postoperative pain (VNRS 1–3)	1–3
Moderate-intensity postoperative pain (VNRS 4–6)	4–6
Severe-intensity postoperative pain (VNRS 7–10)	7–10

*Pain status during mobilization*	
No postoperative pain	0
Low-intensity postoperative pain (VNRS 1–3)	1–3
Moderate-intensity postoperative pain (VNRS 4–6)	4–6
Severe-intensity postoperative pain (VNRS 7–10)	7–10

*General condition status*	
Patient is stating feeling well	0
Patient has side effects apart from pain and nausea vomiting (e.g., sensation of warmth, flushing, itching, constipation, and urine retention)	5
Patient has acute severe circulation abnormalities (blood pressure ≤ 80 or ≥200 mmHg or mean arterial pressure < 50 mmHg and heart rate ≤ 40 or >110)	15^**∗**^
Patient has developed acute severe respiratory abnormalities (unusual respiration or respiration rate < 9 or >20/min, long pauses in breathing, and shallow breathing)	15^**∗**^

^*∗*^Any single score of 15 (on either consciousness, circulation, or respiration) should call for immediate activation of acute assistance with the patient.

**Table 2:** Demographics, anthropometrics, and clinical characteristics of patients (*n* = 679) included in the study at the University Hospital of Astana.

	ESS (*n* = 228)	VNRS (*n* = 227)	Control (*n* = 224)	*P* value
Age: mean ± SD	43.4 ± 16.4	42.4 ± 16.4	44.9 ± 15.8	*P* = 0.72^*∗*^
BMI: mean ± SD	26.8 ± 6.1	26.3 ± 5.6	27 ± 5.9	*P* = 0.39^*∗*^
Gender				
Male: *n* (%)	116 (50.8%)	132 (58.1%)	120 (53.6%)	*P* = 0.28^*∗∗*^
Female: *n* (%)	112 (49.2%)	95 (41.9%)	104 (46.4%)	
ASA classification: *n* (%)				
ASA I	5 (2.2%)	6 (2.6%)	5 (2.2%)	*P* = 0.43^*∗∗*^
ASA II	145 (63.6%)	131 (57.7%)	126 (56.2%)	
ASA III	76 (33.3%)	89 (39.2%)	96 (42.9%)	
ASA IV	2 (0.9%)	1 (0.4%)	0	
Type of surgery: *n* (%)				
Orthopedic	202 (88.6%)	199 (87.6%)	207 (92.4%)	*P* = 0.38^*∗∗*^
Abdominal	10 (4.3%)	14 (6.2%)	9 (4%)	
Vascular	16 (7%)	14 (6.2%)	8 (3.6%)	
Type of anaesthesia: *n* (%)				
Sevo + fentanyl	74 (32.4%)	81 (35.7%)	79 (35.2%)	*P* = 0.79^*∗∗*^
Regional	38 (16.6%)	46 (20.2%)	45 (20%)	
SA ± EDA	102 (44.7%)/4 (1.7%)	93 (41%)/4 (1.8%)	89 (39.7%)/6 (2.6%)	
TIVA	14 (6.1%)	9 (3.9%)	8 (3.6%)	

^*∗*^ANOVA; ^*∗∗*^Chi square analysis. Sevo: sevoflurane; EDA: epidural anaesthesia; SA: spinal anaesthesia; TIVA: total intravenous anaesthesia.

**Table 3:** Demographics, anthropometrics, and clinical characteristics of patients (*n* = 473) included in the study at the University Hospital of Krasnodar.

Variables	ESS (*n* = 181)	VNRS (*n* = 190)	Control (*n* = 102)	*P* value
Age: mean ± SD	55.2 ± 14.7	55.1 ± 15.6	56 ± 14.9	*P* = 0.87^*∗*^
BMI: mean ± SD	28 ± 17	27.8 ± 5.9	25.1 ± 4.5	*P* = 0.08^*∗*^
Gender				
Male: *n* (%)	69 (38.2%)	69 (36.4%)	49 (48%)	*P* = 0.13^*∗∗*^
Female: *n* (%)	112 (61.8%)	121 (63.6%)	53 (51.9%)	
ASA classification: *n* (%)				
ASA I	8 (4.4%)	11 (5.7%)	26 (25.5%)	*P* < 0.0001^*∗*^
ASA II	99 (54.6%)	100 (52.6%)	53 (51.9%)	
ASA III	72 (39.7%)	78 (41%)	22 (21.6%)	
ASA IV	2 (1.1%)	2 (1%)	1 (0.9%)	
Type of surgery: *n* (%)				
Abdominal	115 (63.5%)	125 (65.7%)	61 (59.8%)	*P* = 0.0008^*∗∗*^
Endocrine	29 (16%)	18 (9.4%)	5 (4.9%)	
Gynaecology	19 (10.4%)	9 (4.7%)	12 (11.7%)	
Urology	13 (7.1%)	18 (9.4%)	16 (15.6%)	
Vascular	5 (2.7%)	20 (10.5%)	8 (7.8%)	
Type of anaesthesia: *n* (%)				
Sevo + fentanyl	100 (55.2%)	103 (54.2%)	38 (37.2%)	*P* = 0.0034^*∗∗*^
Sevo + fentanyl + EDA	77 (42.5%)	68 (35.7%)	45 (44.1%)	
SA ± EDA	7 (3.8%)/6 (3.3%)	15 (7.8%)/7 (3.6%)	14 (13.7%)/4 (3.9%)	
TIVA	3 (1.6%)	2 (1%)	5 (4.9%)	

^*∗*^ANOVA; ^*∗∗*^Chi square analysis. Sevo: sevoflurane; EDA: epidural anaesthesia; SA: spinal anaesthesia.; TIVA: total intravenous anaesthesia.

**Table 4:** Pooled demographics, anthropometrics, and clinical characteristics of patients included in the study after laparoscopic cholecystectomy from the university hospitals of Astana and Krasnodar (*n* = 114).

	ESS group (*n* = 36)	VNRS group (*n* = 54)	Control group (*n* = 24)	*P* value
Age: mean ± SD	51.8 ± 14.9	49.7 ± 13.4	56 ± 12.5	*P* = 0.15^*∗*^
BMI: mean ± SD	26.8 ± 4.2	29.2 ± 5.8	27.1 ± 4.5	*P* = 0.06^*∗*^
Gender				
Male: *n* (%)	10 (27.7%)	11 (20.4%)	5 (20.8%)	*P* = 0.61^*∗∗*^
Female: *n* (%)	26 (72.3%)	43 (79.6%)	19 (79.2%)	
ASA classification: *n* (%)				
ASA I	1 (2.7%)	4 (7.4%)	3 (12.5%)	*P* = 0.39^*∗∗*^
ASA II	23 (63.8%)	34 (62.9%)	11 (45.8%)	
ASA III	12 (33.3%)	16 (29.6%)	10 (41.6%)	
Type of anaesthesia: *n* (%)				
Sevo + Fentanyl	36 (100%)	47 (100%)	15 (100%)	

^*∗*^ANOVA; ^*∗∗*^Chi square analysis. Sevo: sevoflurane.
